# Auditory Development between 7 and 11 Years: An Event-Related Potential (ERP) Study

**DOI:** 10.1371/journal.pone.0018993

**Published:** 2011-05-09

**Authors:** Dorothy V. M. Bishop, Mike Anderson, Corinne Reid, Allison M. Fox

**Affiliations:** 1 School of Psychology, University of Western Australia, Perth, Australia; 2 School of Psychology, Murdoch University, Perth, Australia; 3 Department of Experimental Psychology, University of Oxford, Oxford, United Kingdom; 4 Neurocognitive Development Unit, University of Western Australia, Perth, Australia; University of Bern, Switzerland

## Abstract

**Background:**

There is considerable uncertainty about the time-course of central auditory maturation. On some indices, children appear to have adult-like competence by school age, whereas for other measures development follows a protracted course.

**Methodology:**

We studied auditory development using auditory event-related potentials (ERPs) elicited by tones in 105 children on two occasions two years apart. Just over half of the children were 7 years initially and 9 years at follow-up, whereas the remainder were 9 years initially and 11 years at follow-up. We used conventional analysis of peaks in the auditory ERP, independent component analysis, and time-frequency analysis.

**Principal Findings:**

We demonstrated maturational changes in the auditory ERP between 7 and 11 years, both using conventional peak measurements, and time-frequency analysis. The developmental trajectory was different for temporal vs. fronto-central electrode sites. Temporal electrode sites showed strong lateralisation of responses and no increase of low-frequency phase-resetting with age, whereas responses recorded from fronto-central electrode sites were not lateralised and showed progressive change with age. Fronto-central vs. temporal electrode sites also mapped onto independent components with differently oriented dipole sources in auditory cortex. A global measure of waveform shape proved to be the most effective method for distinguishing age bands.

**Conclusions/Significance:**

The results supported the idea that different cortical regions mature at different rates. The ICC measure is proposed as the best measure of ‘auditory ERP age’.

## Introduction

Two contrasting models of auditory maturation between childhood and adulthood are suggested by behavioral and imaging studies. The first is the stability model, which predicts that auditory development is complete by middle childhood. This seems supported by findings that detection of auditory signals and frequency discrimination are near adult-like by 6 years of age [1], [2]. Such stability is consistent with findings that Heschl's gyrus (the site of primary auditory cortex) is functionally mature by 7 years of age [3]. An alternative is the incremental model, which predicts gradual improvement in auditory function from childhood to adulthood. This is supported by evidence that some higher-order auditory functions, such as ability to discriminate speech in noise, continue to develop in the teenage years [4]. Furthermore, alterations in myelination and synaptic pruning in secondary auditory cortex continue well into adolescence [3]. Nevertheless, it has been suggested that at least part of the improvement in auditory discrimination with age could be due to developing use of top-down skills affecting task performance [2] ,[5]. A key question is how far improvement in auditory functioning through childhood is a reflection of non-auditory factors affecting task performance, or whether it is indicative of physiological changes in underlying brain systems.

Auditory event-related potentials (ERPs) can provide complementary information to that from behavioural and imaging studies. However, there have been few developmental studies covering a wide age range of school-aged children. Three of the largest studies, by Ponton et al. [6], [7], [8], Albrecht et al. [9] and Sharma et al. [10] documented substantial changes in the auditory ERP, to click trains, tones and syllables respectively, from early childhood to adolescence, continuing into adulthood. However, inspection of their data suggested relatively little change in waveforms for children between 7 and 11 years. Bishop et al. [11] reanalysed data from Albrecht et al [9] and found that the auditory ERP to simple sounds appeared to follow a step function rather than gradual change, with substantial changes in the observed waveform at the start and end of adolescence. Given that the period from 7 to 11 years is one where there is substantial cognitive growth and brain development, this observation raises questions about the underlying causes and functional significance of changes in the auditory ERP. Before addressing those questions, it seems important, however, to ask how robust is the evidence for a step function. The auditory ERP in children can be strongly influenced by the type of stimulus and rate of stimulus presentation, and developmental trends may also differ depending on the electrode sites from which recordings are taken. The analysis by Bishop et al. [11], though based on a relatively large sample, was restricted to cross-sectional data and focused only on comparisons of waveform shape. Furthermore, the rate of stimulus presentation was relatively rapid, with stimulus-onset asynchrony (SOA) of 1 s. In the current study, we recruited a new sample and employed a longer interval between tones to increase the likelihood of observing an adult-like negativity around 100 ms post-stimulus onset (N1) in the waveform [12].

We also focused specifically on two aspects of the auditory ERP that have been distinguished in the literature and appear to represent activity in parallel auditory pathways [7], [8]. These are components measured in the first 150 ms after presentation of an auditory signal, which are generally regarded as obligatory sensory potentials whose characteristics are determined primarily by physical and temporal characteristics of the stimuli, rather than by their psychological significance to the listener [13]. The first of these, the P1, which peaks around 50 ms in adults, is recorded over a wide frontocentral area. Although P1 is much larger in children than in adults, Bishop et al. [11] found little developmental change in this component before adolescence. The second component, Ta, is a later positivity that is evident at temporal electrode sites. In adults, Ta peaks around 100 ms post-stimulus-onset, and is the first part of the T-complex, described by Wolpaw and Penry [14]. Because it occurs around the same time as the N1 at the vertex, it is sometimes regarded as arising from the same source. However, Wolpaw and Penry noted that the auditory response recorded at temporal electrode sites had a very different morphology from that at the vertex. Electrode location is not a good indicator of the source of the measured activity, and subsequent research has shown that the T-complex represents activity in radially-oriented dipole sources, whereas the vertex response has tangentially-oriented generators [7], [15], both located in auditory cortex in the temporal lobe. Although it has been known about for 35 years, the T-complex has been largely neglected in the literature, perhaps because it is relatively small in adults. However, in children the T-complex is a prominent feature of the auditory ERP [16], [17].

As well as conducting conventional analysis on ERP waveforms, we also used time-frequency analysis. This approach is gaining in popularity as a method for analysing event-related electrophysiological responses [18], because it provides insight into underlying mechanisms that might throw light on developmental change [19]. Time-frequency analysis adopts a radically different perspective on the ERP from the traditional view, where the peaks and troughs in a waveform are treated as signals extracted, by averaging, from a background of noise [18]. The focus of time-frequency analysis is on oscillations, which are readily detected in the EEG when a frequency decomposition is performed. Incoming stimuli can lead to synchronisation of phase of oscillations at a given frequency, and this can be detected by computing phase relations across successive trials. Here we focus on two complementary indices: (i) inter-trial coherence (ITC), a measure of the extent to which phase-locking occurs, and (ii) event-related spectral perturbation (ERSP), a measure of the increase in power in a frequency band after presentation of a signal, relative to baseline. These measures are not just an alternative way of representing data: they are sensitive to features in the data that can get averaged out by conventional methods of analysis [18]. For instance, if an incoming signal leads to a boost in power at a given frequency, but the phase of the response is random, then this would not be detected in an averaged ERP, but would be evident in the ERSP measure, derived from single trials. Similarly, if there is no increase in power when a signal is perceived, but the phase of oscillations is reset, the averaged ERP can give a misleading impression that the response involves additional power, for instance, increased neuronal firing, when the ITC would show instead that the brain oscillations on individual trials have not changed in amplitude, but have rather become aligned in phase to the signal onset. As Klimesch and colleages have noted in the context of visual ERPs [20], if an ERP is generated by an increase in power in response to the stimulus, we might expect to see an increase in phase alignment of the ERP across trials (ITC), but this would necessarily be accompanied by an event-related increase in signal amplitude for individual trials (ERSP). If, on the other hand the grand average ERP is the consequence of phase resetting of ongoing oscillations, we might see increased ITC accompanied by either an increase in power in individual trials, no change in power, or an event-related drop in amplitude (event-related desynchronisation). Furthermore, the pattern of phase synchronisation and amplitude change may vary across frequencies. Therefore we can illuminate underlying mechanisms of ERP generation by studying how ITC and ERSP in different frequency bands relate to the grand average ERP.

The use of time-frequency analysis to investigate development of auditory processing is still in its infancy, but there is already evidence to suggest that changes in ERPs between childhood and adolescence involve an increase in stimulus-induced phase synchronisation [21], [22], [23]. Of particular interest are studies with preadolescent children reporting enhancement of phase-locked responses in the theta range to sounds [24], [25] ,[26]. A similar, though non-significant, trend is apparent in plots shown by Müller et al.[22], with less theta phase-locking for children aged 9–10 years than for those aged 11–12 years.

Several authors have noted the possibility of using ERPs to identify children who have immature or abnormal auditory development. This is of potential value in investigations of the origins of developmental impairments, especially in the area of language [8], [17]. However, in order for auditory ERPs to be clinically useful, we need to know not only what the average developmental trajectory is for the auditory ERP, but also how much variation there is at a given age. One goal of our study was to examine how well one could predict a child's chronological age from a knowledge of the auditory ERP. The previous study by Bishop et al. [11] suggested this may only be possible across very broad age bands. In the current study, we considered whether it would we could identify indicators of auditory ‘brain age’ that would discriminate levels of brain maturity in pre-adolescent school-aged children.

We used a mixed cross-sectional and longitudinal design; this gives greater power to detect developmental change because it controls for within-group variability at a given age. We measured ERPs to pure tones on two occasions separated by two years. Just over half of the children were 7 years initially and 9 years at follow-up, whereas the remainder were 9 years initially and 11 years at follow-up. As well as measuring amplitude of peaks in the waveform, we conducted time-frequency analysis to investigate development of phase-synchronisation in the evoked signal. We then did independent components analysis (ICA) [18] to identify separate sources of observed waveforms, confirming the distinction between two sources for the auditory ERP. Finally, to quantify how far developmental aspects of the ERP could be used to index brain maturation, we analysed waveform shape using methods based on Bishop et al.[11].

### Specific aims and predictions

- To document developmental trajectories for auditory ERPs in children aged 7 to 11 years. We predicted that, in contrast to previous studies, change in the auditory ERP might be detectable across this age range, given the relatively long SOA and more powerful longitudinal design that we adopted.
- To compare developmental trends at temporal vs. frontocentral electrode sites. In line with previous studies we predicted that the signals from these electrode sites have different underlying sources, which would show different developmental trajectories.
- To consider how well the auditory ERP predicted a child's chronological age. We predicted that inclusion of information from time-frequency analysis might give better prediction than reliance on waveform shape alone.

## Materials and Methods

### Ethics statement

The paper reports data from human subjects, and ethical approval was obtained from the University of Western Australia Human Research Ethics Committee. Written informed consent was obtained and the rights of the participants were protected.

### Participants

Children participated in a two-day research program investigating the cognitive, emotional, and social development of children. The program is designed as a child-friendly holiday activity program to enhance task engagement. Children aged 7 or 9 years were recruited during July 2007 and 2008 (initial assessment), and were retested for session 2 during July 2009 and 2010 respectively (follow-up). ERP data were excluded from individuals who were not available for retesting, where a history of neurological disorders or hearing impairment was reported, or where reliable auditory evoked responses were not elicited to the tones (see Fox et al.[27]). The final sample included 62 younger children (31 girls, 31 boys; mean age at initial testing = 7.48 yr, SD = 0.27) and 43 older children (17 girls, 26 boys; mean age at initial testing = 9.49 yr, SD = 0.37).

### Tone stimuli

Auditory stimuli were 1000 Hz sinusoidal tones of 50 ms duration with 2 ms rise and fall times. Sound intensity was calibrated using a 1-second continuous 80 dB SPL tone measured with a Bruel and Kjaer sound level meter.

### Procedure

An electrode cap was fitted and participants were presented with auditory stimuli while they silently read or played electronic games. They were instructed to ignore the tone sequences, but to remain quiet and still throughout the recording session. Stimuli were equiprobable single tones or tone pairs with varying inter-tone interval (25, 50, 100, 200, 400 or 600 ms), presented at random. The interval between trial onsets was 1.5 s and the onset of the first tone was randomly jittered between 0 and 200 ms. For the current analysis, only responses to single tones or to the first tone from pairs with inter-tone interval of 600 ms were studied; these are expected to give similar results, because the auditory ERP is typically complete by 600 ms post-onset. Responses to tone pairs will be reported elsewhere.

### EEG acquisition and analysis

The electroencephalogram (EEG) was recorded continuously (0.5–30 Hz bandpass) from 33 scalp locations referenced to the right mastoid using an electrode cap (EasyCap, Montage 40, excluding TP9 and TP10). Electrodes were also placed above and below the left eye, and on the left mastoid, with an averaged mastoid reference digitally computed offline. Site AFz was used as ground. Data were amplified with a NuAmps 40-channel amplifier, and digitized at a sampling rate of 250 Hz. Offline analysis was performed using SCAN 4.3 and EEGLAB [28].

Ocular artifact reduction was performed on the continuous EEG using regression-based subtraction of the averaged blink artefact identified in the bipolar VEOG channel [29]. Epochs encompassing an interval from 200 ms prior to the onset of the first tone in the pair to 800 ms post-stimulus were extracted and trials contaminated by artifact exceeding ±150 µV were rejected. Averaged waveform analysis was processed with baseline correction from –50 to 0 ms, and data were digitally filtered off-line with a 1-30 Hz, zero phase shift band-pass filter (12 dB down). Automated artefact rejection using higher-order statistics [30] was then applied using default settings in EEGLAB.

### Analytic approach

Results were compared for the two age groups (Younger and Older) at session 1 (2007–2008) and session 2 (2009–2010). Both group and session comparisons are sensitive to changes between 7 and 11 years, but the group comparison is between subjects, whereas the session comparison is within subjects. An interaction between group and session would indicate differing amounts of change from 7 to 9 years than from 9 to 11 years.

### Analysis of mean amplitude of ERP components

Quantitative analyses were conducted on the fronto-central and temporal electrodes (Fz, F3, F4, Cz, C3, C4, T7, T8 and Pz), where the auditory ERP is maximal. Mean amplitude was measured from time windows corresponding to P1 and Ta/N1b regions, as identified previously [27]. The first window, from 58–98 ms corresponds to P1, the second, from 102–146 ms to Ta/N1b. Mean amplitudes were computed for each of these intervals, for each group, session and electrode, and entered into a 3-way ANOVA, with session and electrode as repeated measures, and group as between-subjects factor. Bonferroni adjustment was used to take into account the fact that ANOVAs were run for two intervals, and so a p-value of .025 was regarded as significant. Greenhouse-Geisser correction was applied to correct for violations of sphericity.

### Time-frequency analysis

Time-frequency analysis was then conducted on the specified channels to measure inter-trial coherence (ITC) and event-related spectral perturbation (ERSP). For this analysis, a baseline of 200 ms was used, with frequency extraction using a fast Fourier transform, and a pad ratio of 2. This provides measures of ITC and ERSP in frequency bands with centres at 3.9 Hz, 7.8 Hz, 11.7 Hz, 15.6 Hz and 19.5 Hz. The first band was designated delta, the second of these bands was designated theta, and the third as alpha, the fourth as lower-beta, and fifth as upper-beta. To quantify these results, the mean ITC and mean ERSP were computed over the interval from 100 to 300 ms post-onset. Note that there is a trade-off between time and frequency resolution with time-frequency analysis, and so fairly gross time intervals are used for averaging. To reduce the number of comparisons, data were collapsed across the first three frequency bands, where both the mean ITC values and mean ERSP values showed intercorrelations in excess of .9. Repeated measures ANOVAs were conducted for each frequency band (referred to as 1-3, 4 and 5), with session and electrode as repeated measures, and group as a between subjects factor. To take into account the fact that independent ANOVAs were conducted for three frequency bands, a Bonferroni-corrected value of p = .016 was regarded as significant.

A correlational analysis was conducted to consider how far the mean amplitude of P1 and Ta could be predicted from measures of ITC and ERSP at different ages.

### Source localisation

The ICA extraction routines from EEGLAB [28] were used to identify independent signal sources in the grand averaged ERPs for each group and session. The scalp distributions of components identified by this method typically map on to the projection of a single equivalent brain dipole. The default method in EEGLAB will identify as many components as there are channels, but a specified number of components can be extracted by first running a principal components analysis to reduce the dimensionality of the data. An initial inspection of component structure indicated the same two major components in all age bands, and so the ‘runica’ command was run with specification of two components to be extracted. Because polarity of resulting components is arbitrary, they were inspected and inverted if necessary to ensure the same waveform shape for all groups. The ICA weight matrix obtained from the group's grand averaged auditory ERP was then applied to data from individual participants in that group, to generate waveforms for the two components for all children. To compare age trends for the two components, each component was quantified in terms of mean amplitude over the same time windows as used for P1 and Ta/N1b, and entered into ANOVA, with component, session and time window as repeated measures, and group as a between subjects factor.

Finally, the DIPFIT 2.x routine was applied to the components for each group grand average, to estimate the location of bilaterally symmetric dipole generators using a spherical 4-shell BESA model. Note that we used the default adult head model; it has been argued that this will affect amplitude of source activity but not localisation or orientation of estimated dipoles when applied to children [7], [9].

### Analysis of ‘auditory ERP age’

A final analysis was conducted similar to that done by Bishop et al. [11] using the Fisher-transformed intraclass correlation (ICC) statistic to give an overall measure of similarity between an individual's waveform and the grand mean for each of the age groups over the time window from 0 to 400 ms. Individual waveforms were evaluated in terms of similarity to grand means for 7-, 9- and 11-year-olds using the ICC at each of nine electrodes, F3, Fz, F4, C3, Cz, C4, Pz, T7 and T8 over the interval 0 to 400 ms post-stimulus onset. For each electrode, an age-equivalent was allocated, corresponding to the age group for which the ICC was maximal, i.e. 7, 9 or 11. So for instance, if a child's waveform has an ICC of .7 with the 7-year-old grand average, of .85 with the 9-year-old grand average, and .65 with the 11-year-old grand average, the auditory ERP age would be specified as 9 years. These age-estimates were then averaged across all nine electrodes to give an ‘auditory ERP age’ (AEP-age). These figures were then entered into a repeated-measures ANOVA, with session as repeated measure and group (Younger or Older) as between subjects measure.

## Results

### Analysis of peaks: mean amplitude and latency

**Figure 1:**
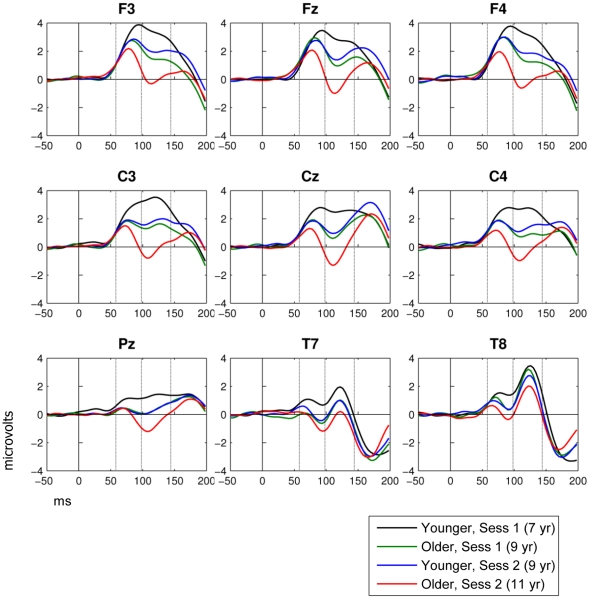
Mean ERP amplitude (µv) by time (ms) at nine sites in relation to group and session. ANOVA confirmed that, in the two windows of interest (demarcated by vertical gray lines), there were substantial age effects on mean amplitudes, as well as significant interactions between age and electrode (see text).

Figure 1 shows the mean waveforms for both groups at sessions 1 and 2. Note that the Younger group at session 2 and the Older group at session 1 are both aged 9 years and their waveforms are similar. This is of interest for two reasons: it demonstrates

replicability of findings across samples, and it also indicates that the auditory ERP is not influenced by prior experience of the task, but rather is a pure index of maturation. Figure 1 also shows the intervals used to define P1 and Ta/N1b. ANOVA output is provided in Table S1 and Table S2.

#### Analysis of P1

There were significant effects of group and session, with η^2^ = .077 and .116 respectively. The main effect of electrode was also significant, but the interactions between electrode and session or group fell short of significance, indicating that change with age was consistent across electrodes.

#### Analysis of Ta/N1b

The interval containing Ta (temporal electrodes) or N1b (fronto-central electrodes) shows substantial developmental change in amplitude, as evidenced by significant effects of both group and session, with effect sizes of η^2^ = .162 and .370 respectively. Both these age factors interacted with electrode, and scrutiny of the means indicates that the greatest decline was seen at the fronto-central electrodes and less at temporal electrodes.

#### Lateralization of ERPs

Scrutiny of Figure 1 suggests there is a marked lateralization of response at temporal electrode sites, but not at frontal electrode sites. Paired t-tests were used to compare left and right-sided mean amplitudes for P1 and Ta/N1b at frontal, central and temporal electrodes for Younger and Older groups at each session. Because 24 pairwise comparisons were conducted, a p-value of .05/24 = .002 was regarded as significant. Results are summarised in Table 1. At frontal electrodes, no lateral comparisons were significant. The amplitude of the temporally distributed positivity (labelled Ta/N1b) was substantially larger at right-sided temporal electrode sites than at left-sided temporal electrode sites. Central electrodes were the only location to show a significant difference for P1, and this only for the Older group in sesssion 1. There was no hint of this effect for the Younger children in session 2, who were also 9 years old, suggesting this might have been a chance finding. The central electrodes also gave a significant lateralisation for Ta/N1b, though of smaller absolute magnitude than for temporal electrodes (at 2 SE difference in means) and, absent for the oldest children (Older group, time 2).

**Table 1:** Mean difference between left- and right-sided electrodes for mean amplitude of P1 and Ta/N1b.

P1			
Younger Group, d.f. = 61			
**Site**	**Mean**	**t**	**P**
Frontal, sess 1	−0.10±0.06	−1.70	.095
Central, sess 1	0.34±0.11	3.11	.003
Temporal, sess 1	−0.23±0.14	−1.70	.094
Frontal, sess 2	−0.12±0.08	−1.39	.170
Central, sess 2	0.08±0.09	0.82	.416
Temporal, sess 2	−0.23±0.13	−1.83	.073
**Older Group, d.f. = 42**			
**Site**	**Mean**	**t**	**P**
Frontal, sess 1	−0.10±0.08	−1.35	.185
Central, sess 1	0.63±0.13	4.76	<.001*
Temporal, sess 1	−0.29±0.14	−2.13	.039
Frontal, sess 2	−0.02±0.07	−0.27	.792
Central, sess 2	0.15±0.07	2.00	.052
Temporal, sess 2	−0.30±0.14	−2.20	.034

Ta/N1b			
Younger Group, d.f. = 61			
**Site**	**Mean**	**t**	**P**
Frontal, sess 1	−0.05±0.09	−0.55	.585
Central, sess 1	0.54±0.13	4.11	<.001*
Temporal, sess 1	−1.15±0.15	−7.49	<.001*
Frontal, sess 2	0.02±0.09	0.18	.859
Central, sess 2	0.45±0.12	3.75	<.001*
Temporal, sess 2	−1.08±0.15	−7.35	<.001*
**Older Group, d.f. = 42**			
**Site**	**Mean**	**t**	**P**
Frontal, sess 1	0.11±0.10	1.05	.299
Central, sess 1	0.73±0.17	4.41	<.001*
Temporal, sess 1	−1.18±0.20	−6.02	<.001*
Frontal, sess 2	0.10±0.07	1.36	.181
Central, sess 2	0.18±0.10	1.76	.086
Temporal, sess 2	−1.15±0.17	−6.68	<.001*

*statistically significant after Bonferroni correction.

### Time-frequency analysis: inter-trial coherence

**Figure 2:**
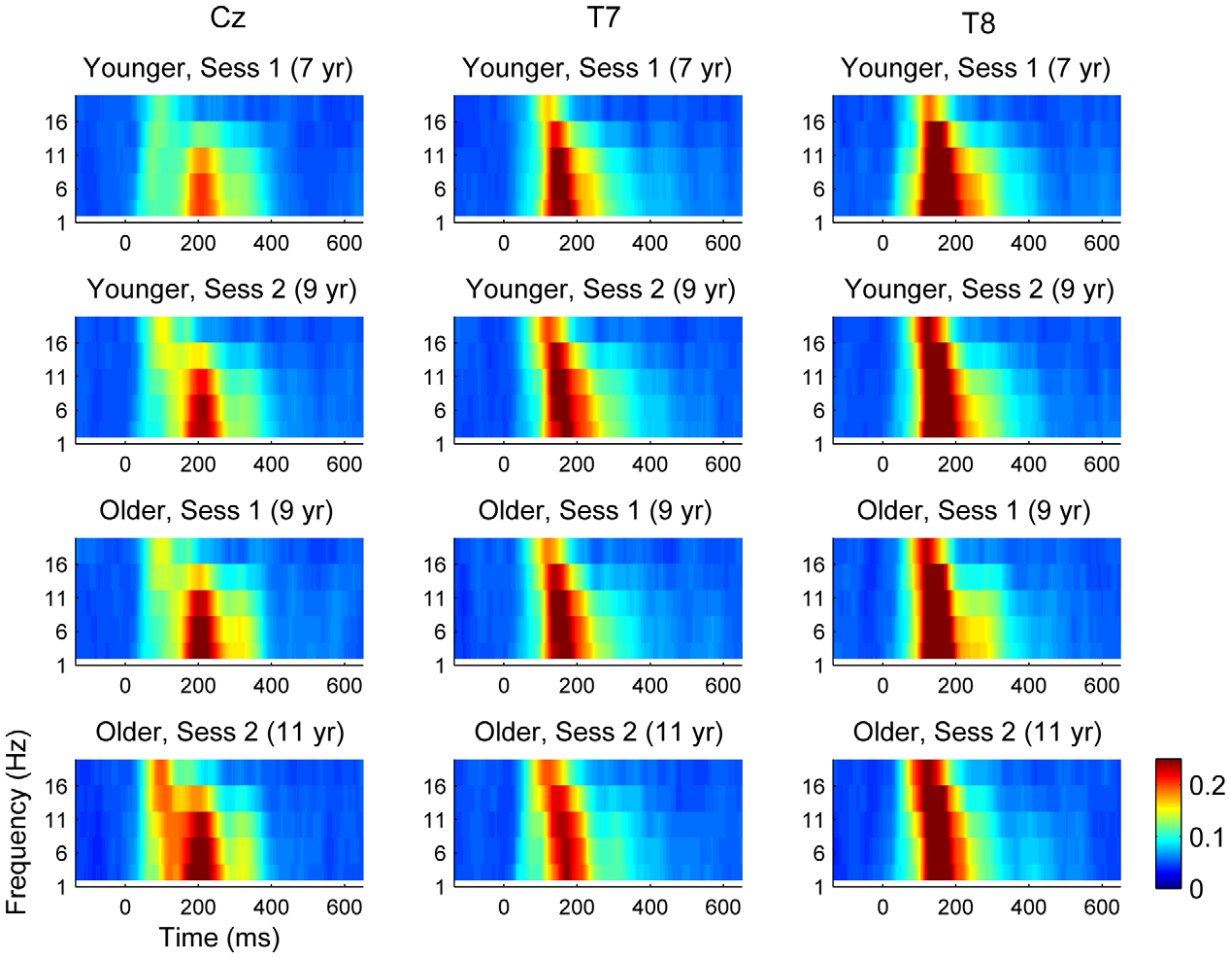
ITC at electrodes Cz, T7 and T8. Mean ITC by time (x-axis) and frequency (y-axis) in relation to group and session. Color bar indicating range from zero, i.e. no synchronization (green) to ITC of 0.2 (deep red). ANOVA indicated that ITC at lower frequencies tends to increase with age at fronto-central electrodes, but remains stable or declines with age at temporal sites.

Plots of ITC for five frequencies in the range 1–20 Hz for electrodes Cz, T7 and T8 are shown for each group and session in Figure 2. These electrodes were selected to illustrate the different patterns seen for temporal electrodes vs. fronto-central electrodes, of which Cz is taken as a representative. For Cz, a developmental trend for increasing ITC with age is visible, especially at the higher frequencies. The temporal electrodes do not appear to show this trend, and there is a marked difference between T7 and T8, with greater ITC on T8 (right temporal). ANOVA output for mean ITC values in the time window 100-300 ms is shown in Table S3. For the lowest frequency band, encompassing delta, theta and alpha, the main effects of session and group were nonsignificant but there was a substantial main effect of electrode, and significant interactions between electrode and session, and electrode and group. The interaction was explored with further ANOVAs looking for session and group effects for individual electrodes. These showed that ITC tended to increase with age for the fronto-central electrodes, but the effect was significant only for Cz, C4 and Pz. ITC remained stable at T7 and showed a significant decline with age at T8. For frequencies in the beta range (Table S4 and Table S5), there were significant effects of session and group, as well as electrode, and no interactions. This indicates that ITC increased with age systematically.

### Time-frequency analysis: event-related spectral perturbation

**Figure 3:**
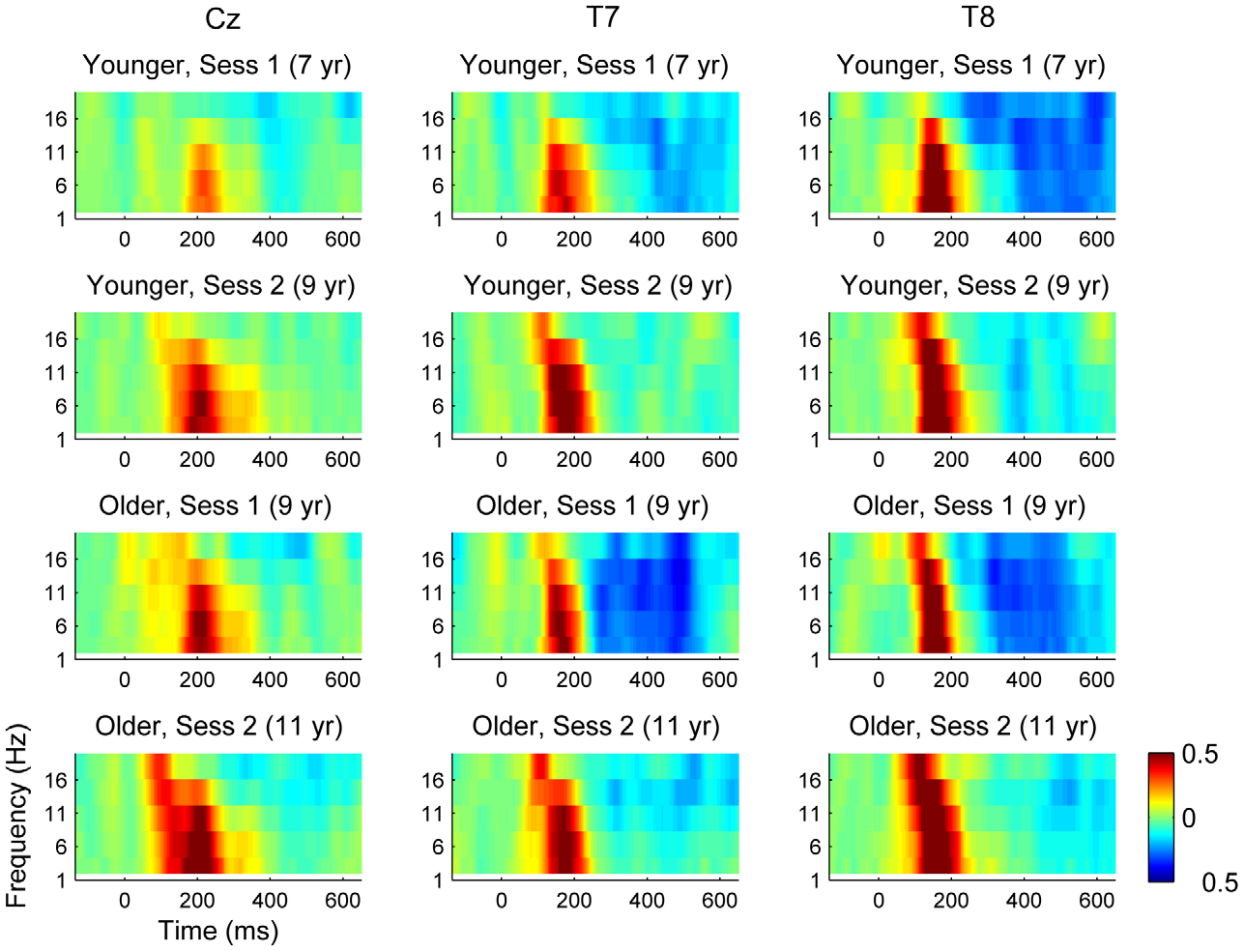
ERSP at electrodes Cz, T7 and T8. Mean ERSP by time (x-axis) and frequency (y-axis) in relation to group and session, with colors indicating range from -.5 (deep blue = power decrease) through zero (green) to .5 (deep red = power increase). ANOVA indicated a general increase in event-related power relative to baseline across all frequencies and all electrodes with age.

Spectral power plots for ERSP are shown for electrodes Cz, T7 and T8 in Figure 3. Visual inspection suggests a trend for increasing power, especially at higher frequencies with age. Note that since ERSP is a measure of power relative to baseline, this could reflect a reduction of noise in the baseline for older children as much as an increase in power post signal onset. Table S6, Table S7 and Table S8 show the results from ANOVA, which was conducted in the same way as for ITC. For each frequency band, there was a significant effect of session. The effect of group tended to fall short of significance, though with a trend in the same direction. There was a main effect of electrode but this did not interact with group or session. The ANOVA thus confirmed a general increase in event-related power relative to baseline across all frequencies and all electrodes with age.

### Time-frequency analysis: correlations among ITC and ERSP and mean amplitudes of P1 and Ta/N1b

Table S9 shows the correlations among ITC, ERSP and mean amplitudes of P1 for different electrodes and frequencies for Younger and Older groups in each session. Table S10 shows analogous information for Ta/N1b. Stringent Bonferroni correction is inappropriate here, because of the high intercorrelations between electrodes and frequencies. For P1, the pattern of correlations shifts with development. The youngest group, 7-year-olds, show significant correlations between P1 amplitude and ITC at certain electrodes and frequencies, with the strongest correlations for electrode T7. Given the latency of P1 at 60-100 ms, we would expect the alpha and low beta frequencies to show strongest relationships with P1 amplitude; while there was variation from electrode to electrode and age to age, the beta-range frequencies at frontal electrodes showed the most consistent correlations with P1. A pattern of more modest correlations between P1 and ITC at frontal electrodes is seen in 9-year-olds, which disappears completely for 11-year-olds. ESRP shows a complementary pattern, with few significant relationships to P1 amplitude for younger children, except at electrode T7, but significant correlations for ERSP at frontal electrodes and C3. It is worth noting that P1 is much smaller in 11-year-olds than 7-year-olds (see Table S1).

For Ta/N1b note that the polarity of the peak is opposite for the fronto-central electrodes and the temporal electrodes. Given the latency of Ta at 100–150 ms, we would expect to see strongest correlations with the theta and alpha frequency ranges. The youngest children, 7-year-olds, show a strong correlation between amplitude of Ta/N1b and ITC at all electrodes except Pz, which is most marked at the lowest frequencies. In contrast, only two correlations with ERSP exceed .3 at this age. These correlations with ITC decline and become less consistent for the two groups of 9-year-olds, though are still evident at some frontal electrodes at lower frequencies, and for electrode T8. Correlations with ERSP are mostly non-significant and not consistent across the two groups of 9-year-olds. For 11-year-olds, there is again a pattern of significant correlations between Ta/N1b amplitude and ITC at lower frequencies, but this is now apparent for Cz, C4 and Pz, and not for frontal or temporal electrodes. Again, there is some indication of a relationship with ERSP at higher frequencies at frontal sites. Once again, note that the size of Ta/N1b is considerably smaller in 11-year-olds than the other groups.

Overall, the pattern of results is consistent with the view that synchronisation of oscillations plays a role in determining the amplitude of both P1 and Ta/N1b in younger children, for whom these peaks are most evident.

### Source localisation

**Figure 4:**
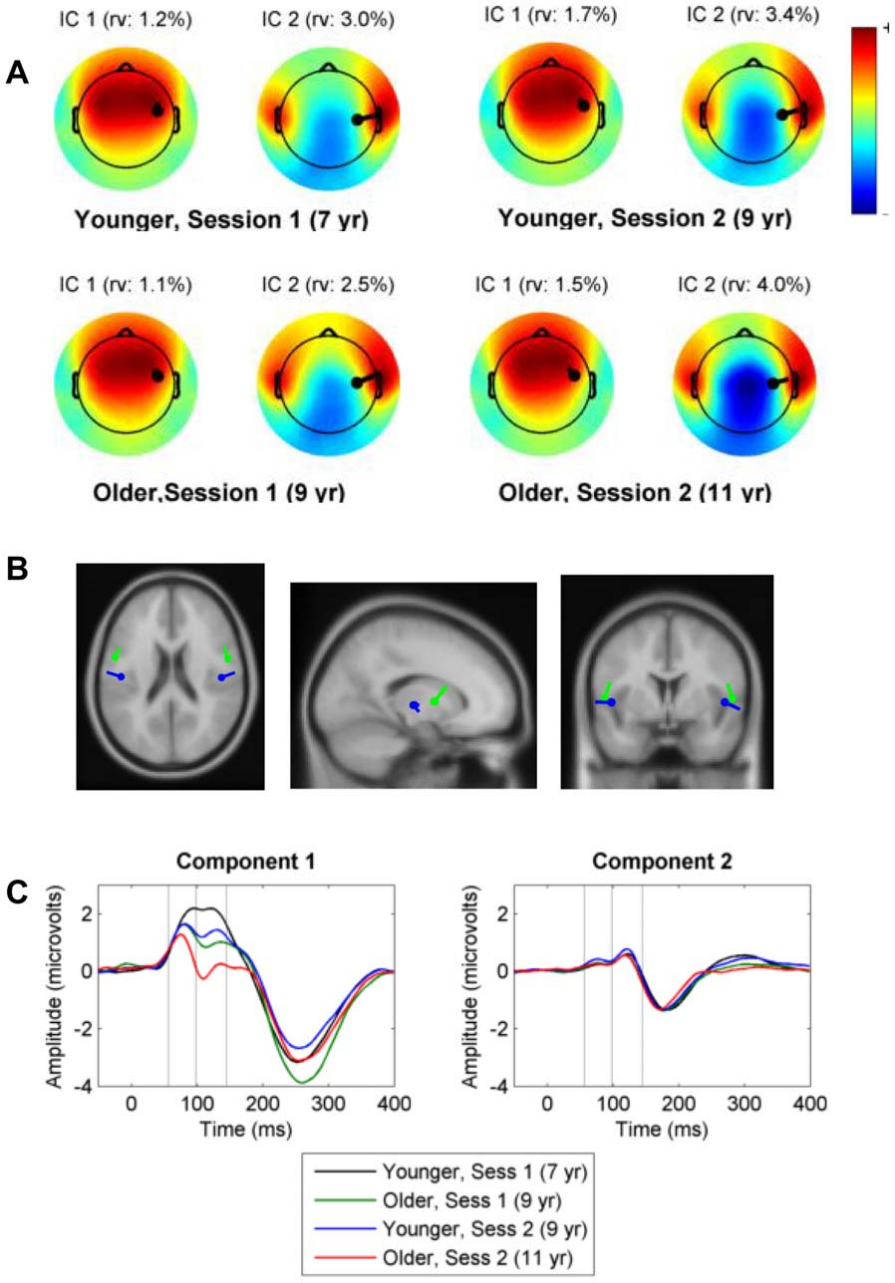
Characteristics of two components identified by ICA. Panel A: Scalp distribution of activity from component 1 (IC 1) and 2 (IC 2) for groups subdivided by age and session. The location of the right-sided dipole is shown in black, and its residual variance (rv) is shown above each plot. (The symmetrical left-sided dipole is not shown). Panel B: Location of dipoles for IC1 (green) and IC2 (blue), based on spherical 4-shell BESA model. Values are derived from grand mean ERP of all groups, since differences between groups were negligible. The MRI used to plot the result is the average MNI (adult) brain. Co-ordinates for dipole 1 are x = 54, y = 0, z = 4; dipole 2: x = 48, y = −12, z = 2. Panel C: Mean component activations for IC1 and IC 2. ANOVA confirmed that, in the two windows of interest (demarcated by vertical gray lines), there were substantial age effects for IC 1, whereas age effects were non-significant for IC 2 (see text).

Figure 4, panel A, shows the scalp distribution for two independent components (IC1 and IC 2) identified for each grand average (age group x session), together with the location of the right-sided dipole for each component. It is evident from inspection that the components are very similar at all ages, with the first one indexing activity recorded from frontocentral channels, and the second indexing activity from temporal electrode sites, with reversal of polarity at central and centro-posterior electrode sites. The dipole locations were closely similar for all four groups, with component 1 being tangential and component 2 radial. Figure 4, panel B, shows the dipole locations in more detail (based on grand means collapsed across all participants), superimposed on a standard MNI template. Both dipoles are located on the superior surface of the temporal lobe. Dipole 1 is located antero-lateral to dipole 2. According to the Jülich Histological Atlas, both dipoles fall within the standard space probabilistic maps for primary auditory cortex based on cytoarchitecture [31].

Components were reconstructed for individual participants using the ICA weights for their age-group and session; Figure 4, panel C shows the mean amplitude for these. A preliminary four-way ANOVA was conducted to compare developmental effects on the two components, with repeated measures of component, window and session, with group as a between-subjects factor. This confirmed a significant interaction between component and session, F (1, 103) = 55.55, p<.001, η^2^ = .34, but the interaction between component and group did not reach significance, F (1, 103) = 1.97, p = .163. Three-way ANOVAs were conducted on P1 and Ta/N1b windows for each component separately to explore the age effects more fully. For component 1 there were substantial age effects reflected in both session, F (1, 103) = 43.8, p<.001, η^2^ = .30, and group factors, F (1, 103) = 20.3, p<.001, η^2^ = .17. In contrast, a parallel ANOVA with component 2 revealed no significant effect of session, F (1, 103) = 1.91, p = .17, or group, F (1, 103) = 3.73, p = .06.

### Use of ICC to estimate ‘auditory ERP age’ for Younger and Older groups at session 1 and session 2

The final analysis considered how far a global measure of waveform similarity, based on the Fisher-transformed ICC, could predict a child's age. There was a significant effect of group, F (1, 105) = 37.2, p<.001, partial η^2^ = .27, and a significant effect of session, F (1, 103) 126.3, p<.001, partial η^2^ = .55, but the interaction fell short of significance. Younger children had a mean AEP-age of 7.94 (SD = 0.61) at session 1 and a mean AEP-age of 8.61 (SD = 1.07) at session 2, (when chronological ages were 7.48 and 9.48 respectively). Older children had a mean AEP-age of 8.71 (SD = 0.82) at session 1, and a mean of 9.51 (SD = 1.21) at session 2, when their chronological ages were 9.49 and 11.49 respectively. Note that AEP age is typically overestimated for the youngest children and underestimated for the oldest children; this is a consequence of the fact that the only possible values for AEP age are 7, 9 and 11 years, and so there is no possibility of obtaining a below-age estimate at 7 years, or an above-age estimate at 11 years.

In a final analysis, we considered whether time-frequency indices increased predictive power when added to a regression equation for predicting chronological age from AEP-age. In a stepwise regression, the AEP-age accounted for the major part of the variance, with adjusted R^2^ = .366, p<.001. The ERSP at T8 in the 4^th^ frequency band was the only time-frequency measure to account for significant additional variance, with R^2^ change = .015, p = .033.

## Discussion

We found more evidence of developmental change in the AEP between 7 and 11 years than was evident in the study of Bishop et al. [11]. There are several possible reasons for the difference, including use of an oddball design with shorter inter-trial interval by Bishop et al. [11]. In addition, the current study had a larger sample size, and used a repeated measures design which has greater power to detect age effects.

The age change in the current study was evident for conventional averaged waveforms from individual electrodes, for independent components derived from these with different dipole orientations and for indices from time-frequency analysis. A global index of waveform similarity to the age mean was effective in predicting a child's age, though much variance remained unexplained, indicating that factors other than chronological age affected ERPs.

There was, however, a clear difference between fronto-central and temporal electrode sites. Overall, our results strongly supported the work of Ponton and colleagues [7], who argued that auditory maturation could not be regarded as a unitary process, because different pathways mature at different rates. We found that at fronto-central electrodes, inter-trial coherence at all frequencies tended to increase with age. As seen in Figure 4, dipole analysis indicated that these electrode sites reflected activity of an underlying tangentially-oriented dipole in auditory cortex. The activity recorded at temporal electrodes, which was the main contributor to our second dipole, radially-oriented in auditory cortex, showed a different pattern. Inter-trial coherence of Ta showed no evidence of increase with age at low frequencies, instead showing a tendency to decline at electrode T8. Another difference between frontal and temporal electrodes was in lateralisation of responses. At temporal electrodes, but not at frontal electrodes, ERPs were lateralised, even though sound presentation was binaural. In adults, a larger T-complex on the right side was noted many years ago by Wolpaw and Penry [32], who suggested this was indicative of a physically larger superior temporal gyrus.

In the introduction, we considered different models of age-related change in the ERP. These results suggest that, over the age range considered here, the stability model fits activity at temporal electrodes, whereas the incremental model is more appropriate for responses measured at fronto-central electrodes. This would be compatible with a view that responses from temporal electrodes reflect activity from neural regions that are largely independent of the components measured at fronto-central electrodes, as suggested by Tonnquist-Uhlen et al. [8], (who used a different nomenclature, so that their electrodes T3 and T4 are comparable to our T7 and T8). Using click train stimuli, they too found little evidence of developmental change at temporal electrodes, whereas fronto-central electrodes showed marked changes with age. A study by Gomes and colleagues [33], however, offers a note of caution: they found no change in amplitude of what they termed ‘central N1’, i.e. a vertex negativity around 100 ms, when a very long SOA (4200 ms) was used and children were required to respond to the tones. While it is possible that their failure to detect an effect resulted from low statistical power (between 10 to 18 participants per age-band), that seems unlikely, given that they did find a trend for an age effect for a later negativity seen around 150 ms with a radial source. They did not analyse positive peaks, so it is not possible to compare their findings at temporal electrodes with those observed here. Nevertheless, their findings suggest that maturational differences between sources may be influenced by stimulus and experimental parameters.

Tonnquist-Uhlen et al. [8], suggested, on the basis of earlier studies of dipole source modeling [7], that activity at electrodes T3 and T4 represent activity in secondary auditory cortices, whereas midline potentials have a contribution from both primary and secondary auditory areas. This conclusion is based on orientation of cortical pyramidal cells in the gyri and sulci of primary and secondary cortex; in particular, radially-oriented generators would reflect activity only from the lateral surface of the temporal lobe [7]. A subsequent study using magnetoencephalography (MEG) found that peak activations at 70 and 100 ms could be localised to different sub-areas of Heschl's gyrus, and showed marked developmental change [34]. Note, however, that the dipole activity underlying the T-complex would not be detected using MEG, which is insensitive to radially-oriented sources. Source localisation analysis of our data supported the existence of separate generators in auditory cortex: a tangentially-oriented one that showed substantial developmental change between 7 and 11 years, and a radially-oriented one that did not show age changes. Overall, our results support those of Tonnquist-Uhlen and colleagues in indicating the independence of different generators of auditory potentials, with the radial dipoles in the lateral temporal lobe (indexed by activity in temporal electrodes) showing stability across age relative to other regions of auditory cortex.

The time-frequency analysis confirms the importance of synchronisation of phase of oscillations with a signal in the generation of the auditory ERP. This showed clear developmental trends over this age range, broadly consistent with phenomena described by previous authors, and confirming the point made by Uhlhass et al. [19] that synchronisation of oscillatory activity is an important index of maturity and efficiency of cortical networks. Note, however, that we found increases in event-related power as well as in phase coherence with age, but in general, the ITC measures of phase coherence were better predictors of mean amplitude of ERP peaks than ERSP. Nevertheless, insofar as our purpose was to find an index that was efficient at distinguishing between age bands, our global measure of waveform shape, the ICC, was the most effective measure. In future work, we plan to consider how this index of auditory maturity relates to behavioral indices.

## Supporting Information

Table S1 ANOVA: P1 mean amplitude.
(DOC)

Table S2 ANOVA: Ta/N1b mean amplitude.
(DOC)

Table S3 ANOVA: mean ITC, frequency band 1–3 (delta, theta, alpha), 100–300 ms.
(DOC)

Table S4 ANOVA: mean ITC, frequency band 4 (lower beta), 100–300 ms.
(DOC)

Table S5 ANOVA: mean ITC,frequency band 5 (upper beta), 100–300 ms.
(DOC)

Table S6 ANOVA: mean ERSP, frequency bands 1–3 (delta, theta, alpha), 100–300 ms.
(DOC)

Table S7 ANOVA: mean ERSP, frequency band 4 (lower beta), 100–300 ms.
(DOC)

Table S8 ANOVA: mean ERSP, frequency band 5 (upper beta), 100–300 ms.
(DOC)

Table S9 Correlations among ITC, ERSP and mean amplitude of P1 for different ages and frequency bands (δ, θ, α, β1, β2).
(DOC)

Table S10 Correlations among ITC, ERSP and mean amplitude of Ta/N1 for different ages and frequency bands (δ, θ, α, β1, β2).
(DOC)
