# Supplementary material for: Auditory Development between 7 and 11 Years: An Event-Related Potential (ERP) Study
Source: PLoS One. 2011 May 9;6(5):e18993. doi: 10.1371/journal.pone.0018993 (PMC3090390; doi:10.1371/journal.pone.0018993)
Supplement: Table S1 — ANOVA: P1 mean amplitude. (DOC) [file pone.0018993.s001.doc]

**Appendix S1**

ANOVA: P1 mean amplitude

| **Between-subject effects** | F | p | partial η^2^ |  |
| --- | --- | --- | --- | --- |
| Group (Younger vs Older) | 8.7 | 0.004 | 0.077 |  |
| **Within-subject effects** |  |  |  |  |
| Session (Time 1 vs Time 2) | 13.5 | <.001 | 0.116 |  |
| Session x Group | 0.0 | 0.84 | 0 |  |
| Electrode | 91.0 | <.001 | 0.469 |  |
| Electrode x Group | 1.2 | 0.306 | 0.011 |  |
| Session x Electrode | 0.3 | 0.97 | 0.003 |  |
| Session x Electrode x Group | 2.6 | 0.032 | 0.024 |  |
|  |  |  |  |  |
| **Mean (SD)** | Younger,  sess 1 | Older,  sess 1 | Younger , sess 2 | Older,  sess 2 |
| F3 | 2.2 (1.80) | 1.5 (1.35) | 1.6 (1.49) | 1.0 (1.40) |
| Fz | 2.0 (1.95) | 1.5 (1.40) | 1.5 (1.54) | 0.8 (1.43) |
| F4 | 2.1 (1.65) | 1.5 (1.37) | 1.6 (1.42) | 0.7 (1.37) |
| C3 | 2.6 (1.97) | 2.1 (1.47) | 2.2 (1.71) | 1.6 (1.31) |
| Cz | 2.3 (2.05) | 2.3 (1.69) | 2.1 (1.79) | 1.5 (1.40) |
| C4 | 2.5 (1.96) | 2.3 (1.43) | 2.3 (1.78) | 1.4 (1.31) |
| Pz | 1.0 (1.64) | 0.5 (1.16) | 0.2 (1.32) | 0.1 (1.23) |
| T7 | 0.9 (1.45) | -0.2 (1.26) | 0.1 (1.17) | -0.4 (1.08) |
| T8 | 1.2 (1.42) | 0.7 (1.31) | 0.7 (1.10) | 0.2 (1.06) |
