# Supplementary material for: Auditory Development between 7 and 11 Years: An Event-Related Potential (ERP) Study
Source: PLoS One. 2011 May 9;6(5):e18993. doi: 10.1371/journal.pone.0018993 (PMC3090390; doi:10.1371/journal.pone.0018993)
Supplement: Table S2 — ANOVA: Ta/N1b mean amplitude. (DOC) [file pone.0018993.s002.doc]

**Appendix S2**

ANOVA: Ta/N1b mean amplitude

| **Between-subject effects** | F | p | partial η2 |  |
| --- | --- | --- | --- | --- |
| Group (Younger vs Older) | 19.9 | <.001 | 0.162 |  |
| **Within-subject effects** |  |  |  |  |
| Session (Time 1 vs Time 2) | 60.5 | <.001 | 0.370 |  |
| Session x Group | 0.3 | 0.564 | 0.003 |  |
| Electrode | 27.0 | <.001 | 0.208 |  |
| Electrode x Group | 5.9 | <.001 | 0.054 |  |
| Session x Electrode | 3.3 | 0.007 | 0.031 |  |
| Session x Electrode x Group | 0.7 | 0.614 | 0.007 |  |
|  |  |  |  |  |
| **Mean (SD)** | Younger,  sess 1 | Older,  sess 1 | Younger , sess 2 | Older,  sess 2 |
| F3 | 3.2 (2.50) | 1.4 (1.96) | 1.8 (2.14) | -0.2 (2.10) |
| Fz | 2.5 (2.65) | 1.2 (2.24) | 1.5 (2.25) | -0.4 (1.99) |
| F4 | 2.6 (2.53) | 0.8 (2.20) | 1.4 (2.28) | -0.3 (2.28) |
| C3 | 3.2 (2.60) | 1.5 (2.12) | 2.0 (2.32) | 0.1 (2.41) |
| Cz | 2.8 (2.60) | 1.3 (2.23) | 1.7 (2.31) | -0.3 (2.29) |
| C4 | 3.1 (2.58) | 1.4 (2.38) | 1.9 (2.50) | -0.2 (2.37) |
| Pz | 1.3 (2.04) | 0.5 (1.75) | 0.3 (1.74) | -0.6 (1.43) |
| T7 | 1.1 (1.88) | 0.2 (1.44) | 0.2 (1.40) | -0.5 (1.52) |
| T8 | 2.5 (1.87) | 2.0 (1.93) | 1.8 (1.71) | 1.1 (1.78) |
