# Supplementary material for: Auditory Development between 7 and 11 Years: An Event-Related Potential (ERP) Study
Source: PLoS One. 2011 May 9;6(5):e18993. doi: 10.1371/journal.pone.0018993 (PMC3090390; doi:10.1371/journal.pone.0018993)
Supplement: Table S3 — ANOVA: mean ITC, frequency band 1–3 (delta, theta, alpha), 100–300 ms. (DOC) [file pone.0018993.s003.doc]

**Appendix S3**

ANOVA: mean ITC, frequency band 1-3 (delta, theta, alpha), 100-300 ms

| **Between-subject effects** | F | p | partial η2 |  |
| --- | --- | --- | --- | --- |
| Group (Younger vs Older) | 2.7 | .102 | 0.026 |  |
| **Within-subject effects** |  |  |  |  |
| Session (Time 1 vs Time 2) | 1.5 | .218 | 0.015 |  |
| Session x Group | 0.7 | .407 | 0.007 |  |
| Electrode | 33.2 | <.001 | 0.244 |  |
| Electrode x Group | 3.5 | .011 | 0.033 |  |
| Session x Electrode | 8.5 | <.001 | 0.076 |  |
| Session x Electrode x Group | 2.4 | .058 | 0.022 |  |
|  |  |  |  |  |
| **Mean (SD)** | Younger,  sess 1 | Older,  sess 1 | Younger , sess 2 | Older,  sess 2 |
| F3 | 0.159 (0.052) | 0.164 (0.047) | 0.154 (0.052) | 0.182 (0.065) |
| Fz | 0.142 (0.039) | 0.166 (0.051) | 0.158 (0.056) | 0.190 (0.065) |
| F4 | 0.149 (0.046) | 0.166 (0.055) | 0.154 (0.058) | 0.181 (0.074) |
| C3 | 0.173 (0.050) | 0.174 (0.052) | 0.166 (0.049) | 0.181 (0.062) |
| Cz | 0.165 (0.048) | 0.176 (0.054) | 0.164 (0.056) | 0.189 (0.064) |
| C4 | 0.176 (0.054) | 0.184 (0.060) | 0.171 (0.055) | 0.189 (0.060) |
| Pz | 0.105 (0.034) | 0.132 (0.054) | 0.126 (0.050) | 0.146 (0.057) |
| T7 | 0.167 (0.066) | 0.170 (0.062) | 0.171 (0.072) | 0.161 (0.070) |
| T8 | 0.215 (0.057) | 0.204 (0.067) | 0.201 (0.076) | 0.191 (0.070) |
