# Supplementary material for: Auditory Development between 7 and 11 Years: An Event-Related Potential (ERP) Study
Source: PLoS One. 2011 May 9;6(5):e18993. doi: 10.1371/journal.pone.0018993 (PMC3090390; doi:10.1371/journal.pone.0018993)
Supplement: Table S5 — ANOVA: mean ITC,frequency band 5 (upper beta), 100–300 ms. (DOC) [file pone.0018993.s005.doc]

**Appendix S5**

ANOVA: mean ITC,frequency band 5 (upper beta), 100-300 ms

| **Between-subject effects** | F | p | partial η2 |  |
| --- | --- | --- | --- | --- |
| Group (Younger vs Older) | 7.1 | .009 | 0.064 |  |
| **Within-subject effects** |  |  |  |  |
| Session (Time 1 vs Time 2) | 36.5 | <.001 | 0.262 |  |
| Session x Group | 0.1 | .785 | 0.001 |  |
| Electrode | 36.5 | <.001 | 0.262 |  |
| Electrode x Group | 0.7 | .607 | 0.006 |  |
| Session x Electrode | 2.7 | .026 | 0.025 |  |
| Session x Electrode x Group | 0.5 | .765 | 0.005 |  |
|  |  |  |  |  |
| **Mean (SD)** | Younger,  sess 1 | Older,  sess 1 | Younger , sess 2 | Older,  sess 2 |
| F3 | 0.087 (0.030) | 0.105 (0.039) | 0.103 (0.038) | 0.123 (0.056) |
| Fz | 0.085 (0.027) | 0.105 (0.041) | 0.104 (0.040) | 0.124 (0.050) |
| F4 | 0.086 (0.03) | 0.110 (0.044) | 0.106 (0.037) | 0.125 (0.062) |
| C3 | 0.083 (0.026) | 0.096 (0.033) | 0.100 (0.042) | 0.112 (0.044) |
| Cz | 0.092 (0.031) | 0.107 (0.040) | 0.113 (0.046) | 0.129 (0.050) |
| C4 | 0.087 (0.026) | 0.101 (0.034) | 0.105 (0.044) | 0.122 (0.049) |
| Pz | 0.070 (0.019) | 0.084 (0.029) | 0.078 (0.031) | 0.091 (0.035) |
| T7 | 0.099 (0.035) | 0.113 (0.047) | 0.121 (0.052) | 0.126 (0.064) |
| T8 | 0.113 (0.041) | 0.132 (0.053) | 0.139 (0.058) | 0.153 (0.067) |
