# Supplementary material for: Auditory Development between 7 and 11 Years: An Event-Related Potential (ERP) Study
Source: PLoS One. 2011 May 9;6(5):e18993. doi: 10.1371/journal.pone.0018993 (PMC3090390; doi:10.1371/journal.pone.0018993)
Supplement: Table S6 — ANOVA: mean ERSP, frequency bands 1–3 (delta, theta, alpha), 100–300 ms. (DOC) [file pone.0018993.s006.doc]

**Appendix S6**

ANOVA: mean ERSP, frequency bands 1-3 (delta, theta, alpha), 100-300 ms

| **Between-subject effects** | F | p | partial η2 |  |
| --- | --- | --- | --- | --- |
| Group (Younger vs Older) | 2.7 | .101 | 0.026 |  |
| **Within-subject effects** |  |  |  |  |
| Session (Time 1 vs Time 2) | 8.8 | .004 | 0.078 |  |
| Session x Group | 0.7 | .389 | 0.007 |  |
| Electrode | 5.4 | <.001 | 0.05 |  |
| Electrode x Group | 2.3 | .043 | 0.022 |  |
| Session x Electrode | 0.7 | .639 | 0.007 |  |
| Session x Electrode x Group | 0.5 | .786 | 0.005 |  |
|  |  |  |  |  |
| **Mean (SD** | Younger,  sess 1 | Older,  sess 1 | Younger , sess 2 | Older,  sess 2 |
| F3 | 0.114 (0.315) | 0.202 (0.335) | 0.122 (0.328) | 0.281 (0.345) |
| Fz | 0.115 (0.281) | 0.233 (0.335) | 0.234 (0.349) | 0.346 (0.339) |
| F4 | 0.133 (0.314) | 0.237 (0.309) | 0.222 (0.325) | 0.328 (0.341) |
| C3 | 0.144 (0.325) | 0.149 (0.374) | 0.166 (0.049) | 0.181 (0.062) |
| Cz | 0.150 (0.302) | 0.229 (0.390) | 0.252 (0.348) | 0.338 (0.329) |
| C4 | 0.185 (0.356) | 0.245 (0.391) | 0.223 (0.381) | 0.357 (0.280) |
| Pz | 0.090 (0.271) | 0.145 (0.313) | 0.127 (0.298) | 0.264 (0.306) |
| T7 | 0.184 (0.365) | 0.149 (0.404) | 0.252 (0.411) | 0.256 (0.401) |
| T8 | 0.285 (0.383) | 0.203 (0.413) | 0.299 (0.442) | 0.350 (0.403) |
