# Supplementary material for: Auditory Development between 7 and 11 Years: An Event-Related Potential (ERP) Study
Source: PLoS One. 2011 May 9;6(5):e18993. doi: 10.1371/journal.pone.0018993 (PMC3090390; doi:10.1371/journal.pone.0018993)
Supplement: Table S7 — ANOVA: mean ERSP, frequency band 4 (lower beta), 100–300 ms. (DOC) [file pone.0018993.s007.doc]

**Appendix S7**

ANOVA: mean ERSP, frequency band 4 (lower beta), 100-300 ms

| **Between-subject effects** | F | p | partial η2 |  |
| --- | --- | --- | --- | --- |
| Group (Younger vs Older) | 5.9 | .016 | 0.055 |  |
| **Within-subject effects** |  |  |  |  |
| Session (Time 1 vs Time 2) | 21.6 | <.001 | 0.173 |  |
| Session x Group | 0 | .962 | 0 |  |
| Electrode | 35.2 | <.001 | 0.255 |  |
| Electrode x Group | 1.4 | .233 | 0.014 |  |
| Session x Electrode | 1.5 | .205 | 0.014 |  |
| Session x Electrode x Group | 1.1 | .374 | 0.01 |  |
|  |  |  |  |  |
| **Mean (SD) mean amplitude** | Younger,  sess 1 | Older,  sess 1 | Younger , sess 2 | Older,  sess 2 |
| F3 | 0.114 (0.033) | 0.132 (0.037) | 0.126 (0.046) | 0.154 (0.060) |
| Fz | 0.111 (0.030) | 0.136 (0.047) | 0.135 (0.055) | 0.161 (0.060) |
| F4 | 0.114 (0.036) | 0.138 (0.051) | 0.133 (0.052) | 0.154 (0.066) |
| C3 | 0.120 (0.030) | 0.129 (0.036) | 0.130 (0.049) | 0.145 (0.053) |
| Cz | 0.124 (0.034) | 0.142 (0.049) | 0.141 (0.058) | 0.162 (0.058) |
| C4 | 0.124 (0.033) | 0.139 (0.044) | 0.136 (0.053) | 0.154 (0.052) |
| Pz | 0.087 (0.026) | 0.109 (0.042) | 0.104 (0.043) | 0.119 (0.047) |
| T7 | 0.132 (0.048) | 0.141 (0.057) | 0.151 (0.067) | 0.151 (0.071) |
| T8 | 0.155 (0.043) | 0.167 (0.059) | 0.169 (0.066) | 0.181 (0.069) |
