# Supplementary material for: Auditory Development between 7 and 11 Years: An Event-Related Potential (ERP) Study
Source: PLoS One. 2011 May 9;6(5):e18993. doi: 10.1371/journal.pone.0018993 (PMC3090390; doi:10.1371/journal.pone.0018993)
Supplement: Table S8 — ANOVA: mean ERSP, frequency band 5 (upper beta), 100–300 ms. (DOC) [file pone.0018993.s008.doc]

**Appendix S8**

ANOVA: mean ERSP, frequency band 5 (upper beta), 100-300 ms

| **Between-subject effects** | F | p | partial η2 |  |
| --- | --- | --- | --- | --- |
| Group (Younger vs Older) | 4.2 | .043 | 0.039 |  |
| **Within-subject effects** | F | p | partial η2 |  |
| Session (Time 1 vs Time 2) | 7.6 | .007 | 0.069 |  |
| Session x Group | 0 | .970 | 0 |  |
| Electrode | 4.5 | .001 | 0.042 |  |
| Electrode x Group | 1.3 | .254 | 0.013 |  |
| Session x Electrode | 1.4 | .207 | 0.014 |  |
| Session x Electrode x Group | 0.4 | .855 | 0.004 |  |
|  |  |  |  |  |
| **Mean (SD) mean amplitude** | Younger,  sess 1 | Older,  sess 1 | Younger , sess 2 | Older,  sess 2 |
| F3 | 0.003 (0.338) | 0.112 (0.366) | 0.026 (0.313) | 0.169 (0.335) |
| Fz | 0.042 (0.276) | 0.162 (0.345) | 0.147 (0.293) | 0.236 (0.343) |
| F4 | 0.033 (0.252) | 0.185 (0.331) | 0.148 (0.351) | 0.231 (0.367) |
| C3 | 0.025 (0.290) | 0.075 (0.360) | 0.079 (0.359) | 0.141 (0.311) |
| Cz | 0.057 (0.273) | 0.145 (0.378) | 0.168 (0.334) | 0.227 (0.318) |
| C4 | 0.054 (0.259) | 0.166 (0.331) | 0.087 (0.383) | 0.249 (0.289) |
| Pz | 0.026 (0.279) | 0.090 (0.309) | 0.062 (0.249) | 0.151 (0.272) |
| T7 | 0.037 (0.386) | 0.069 (0.426) | 0.168 (0.418) | 0.160 (0.493) |
| T8 | 0.062 (0.392) | 0.118 (0.405) | 0.214 (0.458) | 0.299 (0.403) |
