# Supplementary material for: Auditory Development between 7 and 11 Years: An Event-Related Potential (ERP) Study
Source: PLoS One. 2011 May 9;6(5):e18993. doi: 10.1371/journal.pone.0018993 (PMC3090390; doi:10.1371/journal.pone.0018993)
Supplement: Table S9 — Correlations among ITC, ERSP and mean amplitude of P1 for different ages and frequency bands (δ, θ, α, β1, β2). (DOC) [file pone.0018993.s009.doc]

Appendix S9

Correlations* among ITC, ERSP and mean amplitude of P1 for different ages and frequency bands (δ, θ, α, β1, β2)

| Correlation of ITC with mean amplitude | | | | | | | | | | | | | | | | | | | | |
| --- | --- | --- | --- | --- | --- | --- | --- | --- | --- | --- | --- | --- | --- | --- | --- | --- | --- | --- | --- | --- |
|  | Younger, session 1 (7 yr) | | | | | Older, session 1 (9 yr) | | | | | Younger, session 2 (9 yr) | | | | | Older, session 2 (11 yr) | | | | |
|  | δ | θ | α | β1 | β2 | δ | θ | α | β1 | β2 | δ | θ | α | β1 | β2 | δ | θ | α | β1 | β2 |
| F3 | .21 | .22 | .27 | **.44** | **.32** | **.35** | **.35** | **.37** | **.37** | .17 | .20 | .21 | .28 | **.37** | **.39** | .09 | .09 | .09 | .09 | .06 |
| FZ | .22 | .24 | .28 | **.38** | **.31** | .23 | .24 | .25 | .22 | .04 | .20 | .23 | .27 | **.31** | **.32** | .03 | .03 | .02 | .00 | .01 |
| F4 | **.30** | **.32** | **.36** | **.42** | **.35** | **.47** | **.48** | **.47** | **.35** | .07 | .20 | .22 | .28 | **.30** | .24 | .18 | .18 | .15 | .08 | .00 |
| C3 | .19 | .20 | .23 | **.32** | .29 | .26 | .26 | .23 | .14 | .06 | .07 | .08 | .12 | .22 | **.34** | -.02 | -.01 | .01 | .09 | .16 |
| CZ | .01 | .02 | .05 | .13 | .12 | -.03 | -.01 | .00 | -.04 | -.03 | .02 | .04 | .09 | .15 | .14 | -.12 | -.12 | -.11 | -.08 | -.04 |
| C4 | -.03 | -.02 | .04 | .11 | .11 | .10 | .11 | .11 | .04 | .03 | .14 | .16 | .21 | **.30** | .24 | -.12 | -.12 | -.11 | -.09 | -.04 |
| PZ | -.02 | -.01 | .01 | .07 | -.07 | -.21 | -.18 | -.13 | -.05 | .05 | .04 | .06 | .10 | .13 | .03 | -.15 | -.13 | -.10 | -.05 | .05 |
| T7 | **.63** | **.61** | **.54** | **.40** | .27 | .12 | .07 | -.02 | -.09 | -.15 | .21 | .20 | .17 | .13 | .07 | .03 | -.01 | -.10 | -.24 | **-.30** |
| T8 | .23 | .23 | .21 | .21 | .27 | -.09 | -.10 | -.11 | -.14 | -.11 | .03 | .02 | .03 | .09 | .15 | -.06 | -.05 | -.07 | -.13 | -.15 |
| Correlation of ERSP with mean amplitude | | | | | | | | | | | | | | | | | | | | |
|  | Younger, session 1 (7 yr) | | | | | Older, session 1 (9 yr) | | | | | Younger, session 2 (9 yr) | | | | | Older, session 2 (11 yr) | | | | |
|  | δ | θ | α | β1 | β2 | δ | θ | α | β1 | β2 | δ | θ | α | β1 | β2 | δ | θ | α | β1 | β2 |
| F3 | .10 | .09 | .03 | -.15 | -.29 | .10 | .10 | .06 | .02 | .04 | .12 | .13 | .16 | .16 | .01 | **.46** | **.45** | **.39** | .23 | .01 |
| FZ | .15 | .14 | .09 | -.07 | -.15 | .11 | .12 | .11 | .11 | .14 | .10 | .10 | .11 | .12 | .08 | **.38** | **.39** | **.34** | .22 | .12 |
| F4 | .20 | .19 | .12 | -.12 | -.27 | .27 | .28 | .28 | .23 | .08 | .27 | .26 | .24 | .20 | .14 | .27 | **.30** | .25 | .19 | .20 |
| C3 | .01 | .00 | -.07 | -.18 | -.19 | -.06 | -.07 | -.15 | -.20 | -.13 | .21 | .02 | .01 | .02 | .06 | **.35** | **.37** | **.35** | .27 | .16 |
| CZ | .12 | .11 | .03 | -.12 | -.14 | -.02 | -.03 | -.04 | -.05 | -.06 | -.04 | -.03 | -.02 | .01 | -.04 | .17 | .15 | .07 | -.06 | -.14 |
| C4 | .26 | .25 | .22 | .05 | -.16 | -.04 | -.02 | -.03 | -.06 | -.08 | .16 | .17 | .18 | .14 | .02 | .14 | .14 | .09 | .04 | .05 |
| PZ | -.10 | -.09 | -.14 | -.21 | -.18 | -.11 | -.10 | -.07 | -.01 | -.01 | .26 | .26 | .23 | .13 | .03 | .01 | .02 | -.01 | -.08 | -.13 |
| T7 | **.36** | **.36** | **.34** | .24 | .18 | -.16 | -.18 | -.24 | **-.31** | **-.34** | **.35** | **.33** | .28 | .16 | .10 | .08 | .06 | .04 | .04 | .08 |
| T8 | .00 | .02 | .00 | .00 | .03 | -.26 | -.26 | -.22 | -.11 | .01 | .07 | .08 | .06 | .11 | .19 | .05 | .02 | -.05 | -.06 | .04 |

*Correlations reaching uncorrected significance level of .05, .01 and .001 respectively: Younger .25, .33, .41; Older .30, .39, .48. Correlations with absolute value greater than or equal to .3 are bolded.
