# Supplementary material for: Auditory Development between 7 and 11 Years: An Event-Related Potential (ERP) Study
Source: PLoS One. 2011 May 9;6(5):e18993. doi: 10.1371/journal.pone.0018993 (PMC3090390; doi:10.1371/journal.pone.0018993)
Supplement: Table S10 — Correlations among ITC, ERSP and mean amplitude of Ta/N1 for different ages and frequency bands (δ, θ, α, β1, β2). (DOC) [file pone.0018993.s010.doc]

Appendix S10

Correlations among ITC, ERSP and mean amplitude of Ta/N1 for different ages and frequency bands (δ, θ, α, β1, β2)

| Correlation of ITC with mean amplitude | | | | | | | | | | | | | | | | | | | | |
| --- | --- | --- | --- | --- | --- | --- | --- | --- | --- | --- | --- | --- | --- | --- | --- | --- | --- | --- | --- | --- |
|  | Younger, session 1 (7 yr) | | | | | Older, session 1 (9 yr) | | | | | Younger, session 2 (9 yr) | | | | | Older, session 2 (11 yr) | | | | |
|  | δ | θ | α | β1 | β2 | δ | θ | α | β1 | β2 | δ | θ | α | β1 | β2 | δ | θ | α | β1 | β2 |
| F3 | **.70** | **.69** | **.63** | **.34** | -.06 | **.41** | **.39** | **.30** | .05 | -.17 | **.42** | **.38** | .25 | .04 | -.05 | .12 | .08 | -.07 | -.28 | **-.37** |
| FZ | **.71** | **.70** | **.63** | .29 | -.18 | .08 | .04 | -.09 | **-.30** | **-.32** | .29 | .25 | .12 | -.06 | -.12 | -.11 | -.13 | -.23 | **-.38** | **-.40** |
| F4 | **.79** | **.78** | **.72** | **.42** | -.01 | .29 | .26 | .14 | -.15 | -.22 | **.42** | **.37** | .23 | -.02 | -.11 | .05 | .01 | -.11 | -.26 | -.23 |
| C3 | **.65** | **.64** | **.59** | **.38** | -.03 | .29 | .27 | .16 | -.12 | -.25 | .24 | .23 | .15 | .01 | -.11 | -.11 | -.11 | -.16 | -.22 | -.18 |
| CZ | **.52** | **.48** | **.34** | -.06 | **-.40** | -.12 | -.14 | -.24 | **-.38** | **-.33** | .10 | .08 | .01 | -.12 | -.18 | **-.40** | **-.41** | **-.43** | **-.45** | **-.39** |
| C4 | **.54** | **.51** | **.38** | .09 | -.19 | -.11 | -.15 | -.26 | **-.37** | -.26 | .07 | .05 | -.02 | -.15 | -.17 | **-.40** | **-.41** | **-.45** | **-.47** | **-.33** |
| PZ | .20 | .15 | .03 | -.15 | **-.33** | **-.31** | **-.30** | **-.32** | **-.32** | -.19 | -.08 | -.08 | -.11 | -.17 | -.22 | **-.43** | **-.43** | **-.46** | **-.45** | -.28 |
| T7 | **.45** | **.42** | **.36** | .18 | -.06 | .09 | .09 | .14 | .19 | .18 | .16 | .15 | .09 | -.02 | -.17 | -.19 | -.21 | -.21 | -.15 | -.04 |
| T8 | **.65** | **.62** | **.58** | **.42** | .15 | **.51** | **.50** | **.45** | .29 | .04 | **.38** | **.36** | **.32** | .27 | .20 | .28 | .25 | .18 | .05 | -.10 |
| Correlation of ERSP with mean amplitude | | | | | | | | | | | | | | | | | | | | |
|  | Younger, session 1 (7 yr) | | | | | Older, session 1 (9 yr) | | | | | Younger, session 2 (9 yr) | | | | | Older, session 2 (11 yr) | | | | |
|  | δ | θ | α | β1 | β2 | δ | θ | α | β1 | β2 | δ | θ | α | β1 | β2 | δ | θ | α | β1 | β2 |
| F3 | .22 | .21 | .07 | -.14 | -.09 | -.06 | -.08 | -.21 | **-.43** | **-.52** | .10 | .09 | .05 | .01 | .00 | .06 | .04 | -.07 | -.23 | -.21 |
| FZ | .16 | .15 | .02 | -.20 | -.12 | -.16 | -.17 | -.25 | **-.36** | **-.38** | .10 | .10 | .05 | -.02 | -.05 | -.23 | -.25 | **-.35** | **-.44** | **-.42** |
| F4 | **.34** | **.33** | .21 | -.10 | -.18 | .09 | .07 | -.05 | -.29 | **-.36** | .11 | .11 | .08 | .03 | .03 | -.19 | -.20 | -.29 | **-.34** | -.21 |
| C3 | .08 | .07 | -.07 | -.24 | -.20 | -.04 | -.03 | -.05 | -.14 | -.25 | -.11 | -.16 | -.19 | -.21 | -.10 | -.14 | -.15 | -.22 | -.28 | -.23 |
| CZ | .11 | .10 | .00 | -.13 | -.05 | -.11 | -.12 | -.16 | -.21 | -.23 | -.08 | -.07 | -.06 | -.07 | -.06 | -.12 | -.12 | -.17 | -.22 | -.24 |
| C4 | .00 | -.01 | -.10 | -.22 | -.11 | -.09 | -.09 | -.15 | -.22 | -.21 | -.13 | -.13 | -.10 | -.09 | -.12 | -.23 | -.23 | -.26 | -.28 | -.26 |
| PZ | .12 | .11 | .04 | -.05 | .02 | -.20 | -.18 | -.18 | -.18 | -.17 | -.03 | -.02 | -.02 | -.03 | -.06 | -.01 | -.01 | -.03 | -.02 | .01 |
| T7 | .13 | .12 | .07 | -.05 | -.09 | .03 | .03 | .03 | .01 | -.01 | .16 | .15 | .14 | .07 | .02 | -.08 | -.07 | -.03 | .04 | .15 |
| T8 | .20 | .18 | .06 | -.10 | -.10 | .16 | .15 | .09 | -.03 | -.11 | **.33** | **.32** | **.30** | .26 | .20 | -.07 | -.09 | -.16 | -.21 | -.17 |

*Correlations reaching uncorrected significance level of .05, .01 and .001 respectively: Younger .25, .33, .41; Older .30, .39, .48. Correlations with absolute value greater than or equal to .3 are bolded.
